# MicroRNA Expression Profiles in Superficial Esophageal Squamous Cell Carcinoma before Endoscopic Submucosal Dissection: A Pilot Study

**DOI:** 10.3390/ijms22094789

**Published:** 2021-04-30

**Authors:** Shintaro Fujihara, Hideki Kobara, Noriko Nishiyama, Kayo Hirose, Hisakazu Iwama, Tsutomu Masaki

**Affiliations:** 1 Department of Gastroenterology and Neurology, Faculty of Medicine, Graduate School of Medicine, Kagawa University, 1750-1 Ikenobe, Miki-cho, Kita-gun, Kagawa 761-0793, Japan; kobara@med.kagawa-u.ac.jp (H.K.); n-nori@med.kagawa-u.ac.jp (N.N.); mizuyou1@med.kagawa-u.ac.jp (K.H.); tmasaki@med.kagawa-u.ac.jp (T.M.); 2 Life Science Research Center, Kagawa University, 1750-1 Ikenobe, Miki-cho, Kita-gun, Kagawa 761-0793, Japan; iwama@med.kagawa-u.ac.jp

**Keywords:** esophageal squamous cell carcinoma, microRNA, endoscopy

## Abstract

Esophageal squamous cell carcinoma (ESCC) has a poor prognosis when diagnosed at an advanced stage, and early detection and treatment are essential to improve survival. However, intraobserver and interobserver variation make the diagnosis of superficial ESCC difficult, and suitable biomarkers are urgently needed. Here, we compared the microRNA (miRNA) expression profiles of superficial ESCC tissues and adjacent normal tissues obtained immediately before esophageal endoscopic submucosal dissection. We found that ESCC and normal tissues differed in their miRNA expression profiles. In particular, miR-21-5p and miR-146b-5p were significantly upregulated and miR-210-3p was significantly downregulated in tumor tissues compared with normal tissues. We also detected significant associations between miRNA expression and ESCC invasion depth and lymphovascular invasion. The same differential expression of miR-21-5p, miR-146b-5p, and miR-210-3p was detected in ESCC cell lines compared with normal esophageal epithelial cells in vitro. However, transfection of ESCC cells with miR-210-3p and miR-21-5p mimics or inhibitors had partial effects on cell proliferation and invasion in vitro. These results indicate that miRNA expression is significantly deregulated in superficial ESCC, and suggest that the potential contribution of differentially expressed miRNAs to the malignant phenotype should be further investigated.

## 1. Introduction

Esophageal carcinoma is the seventh most common cancer worldwide and the sixth leading cause of cancer-related deaths [1]. Esophageal squamous cell carcinoma (ESCC) is the main histopathologic subtype of esophageal cancer, accounting for about 90% of all cases. The 5-year survival rate of patients with late-stage ESCC is less than 15%; however, this may reach 85% among patients diagnosed at earlier disease stages, highlighting the need for early detection and prompt treatment [2,3]. Esophagogastroduodenoendoscopy is the most sensitive method and the gold standard for diagnosis of esophageal cancer and precancerous lesions [4]. Diagnosis of precancerous and superficial ESCC remains challenging for endoscopists because the lesions are easily overlooked by conventional white light endoscopy (WLE); indeed, about 40% of lesions are thought to be missed [5]. WLE endoscopy together with magnifying narrow-band imaging (M-NBI) has thus become the standard method for the detection of superficial ESCC [6,7]. Even so, the invasion depth of ESCC is difficult to determine accurately and intraobserver and interobserver variation are common. Estimates of the accuracy of invasion depth at diagnosis vary between 53% and 78% for WLE and between 57% and 84% for WLE with M-NBI [8,9]. There are other limitations to diagnostic endoscopy, including the difficulty in evaluating lymphovascular invasion using endoscopic techniques, the invasiveness of the procedure, and the requirement for highly trained physicians and expensive equipment [10]. Therefore, there is an urgent need to identify better adjunct diagnostic biomarkers for superficial ESCC.

MicroRNAs (miRNAs) are short (17–25 nucleotides), noncoding, single-stranded RNA molecules that play pivotal roles in the post-transcriptional regulation of gene expression, including genes involved in such crucial cellular processes as proliferation and cell cycle control. Not surprisingly, dysregulation of miRNA expression and/or function occurs in many cancers, and specific miRNAs have been shown to contribute to oncogenesis, epithelial–mesenchymal transition, tumor metastasis, and drug resistance [11,12]. The potential utility of miRNAs as early diagnostic biomarkers has been investigated in many cancers. However, to date, only a few studies have investigated the miRNA profiles of esophageal cancer tissues [13,14,15,16]. Moreover, none of the previous studies have analyzed the miRNA expression profile of superficial ESCC before endoscopic submucosal dissection (ESD), primarily because of sampling difficulties. Biopsy tends to induce mucosal ulceration and may cause scarring changes over time, which in turn could lead to submucosal fibrosis [17] and associated bleeding and perforation. Therefore, it is difficult to obtain a large number of biopsy specimens from patients before ESD for translational research.

Here, we sought to identify differentially expressed miRNAs as potential biomarkers for the detection of superficial ESCC and to examine the effects of selected miRNAs on the growth of ESCC cell lines in vitro.

## 2. Results

### 2.1. Patient Clinicopathological Features

**Figure 1:**
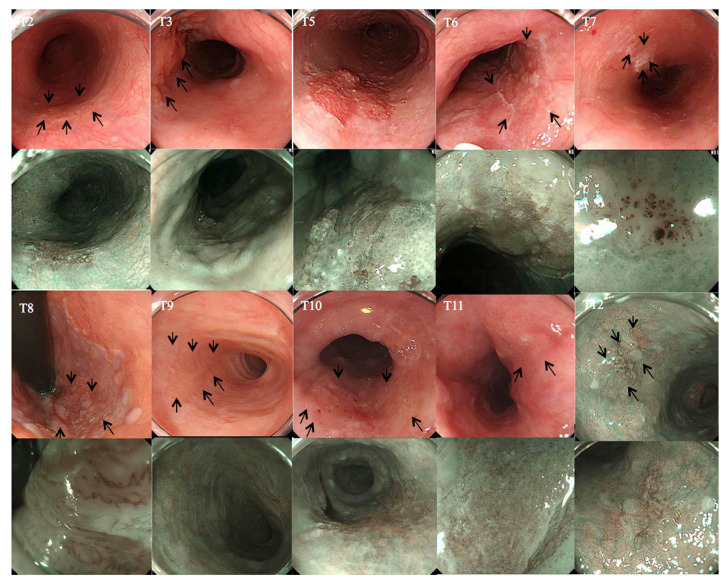
Endoscopic images. Upper: macroscopic appearance of the lesion recorded by white light endoscopy (black arrow). Lower: part of the same lesion observed by narrow-band imaging (NBI) or magnifying narrow-band imaging (M-NBI).

Ten of the 12 pairs of tissue samples contained sufficient material for miRNA analysis. None of the procedures resulted in esophageal perforation that would necessitate endoscopic or surgical hemostasis, blood transfusion, or hospitalization. The baseline characteristics of the 10 patients are shown in Table 1. Their median age was 73.5 years (range 62–82 years) and 8 were male. The median tumor size was 23.5 mm (range 10–48 mm). The endoscopic images of all cases are presented in Figure 1. Five cases were diagnosed as pT1a-epithelium (EP) without lymphovascular invasion. There was a discrepancy between clinical and pathological diagnosis for five patients. For data analysis, patients were also assigned to two invasion depth subgroups: EP (*n* = 5, one patient with high-grade intraepithelial neoplasia was included in this group) and lamina propria mucosa (LP), muscularis mucosae (MM), or superficial submucosa (SM1) (*n* = 5); and two lymphovascular invasion subgroups: negative (*n* = 8) and positive (*n* = 2).

### 2.2. miRNA Expression Analysis

**Figure 2:**
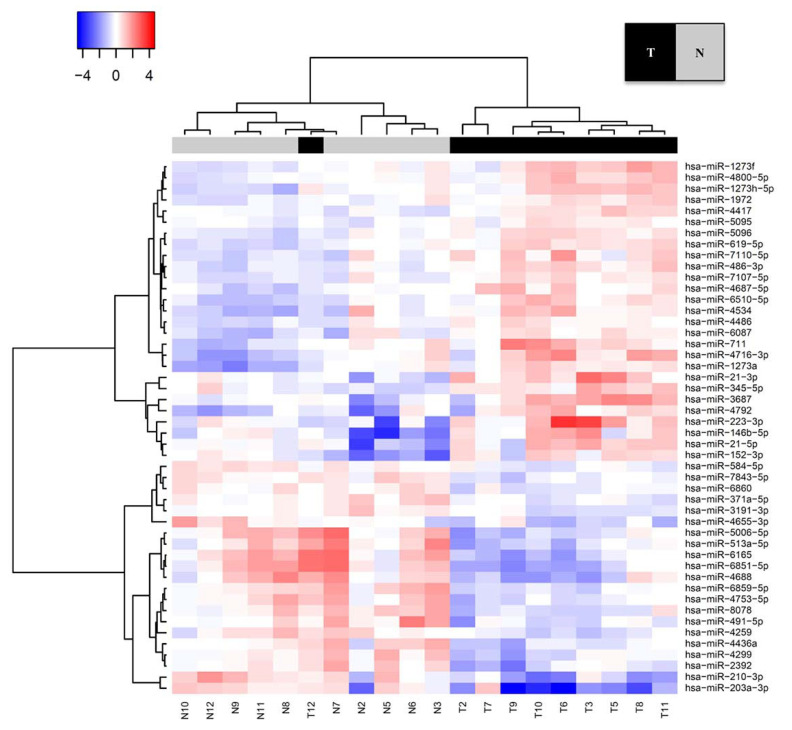
Unsupervised hierarchical clustering analysis of differentially expressed miRNAs between superficial esophageal squamous cell carcinoma (ESCC) and adjacent normal tissues. Columns represent the patients and rows represent the individual miRNAs. Red and blue indicate high and low expression levels, respectively.

We first screened for differentially expressed miRNA in pairs of ESCC and adjacent normal tissues by microarray analysis. Unsupervised hierarchical clustering analysis revealed differential expression between the two tissue groups, and we identified 48 differently expressed miRNAs among the 2555 miRNAs examined (Figure 2). Of these, 27 miRNAs were significantly upregulated and 21 miRNAs were significantly downregulated in ESCC tissues compared with adjacent normal tissues (Table 2). The most highly differentially expressed clusters were all oncomiRs and included miR-146b-5p, miR-21-5p, and miR-210-3p. The expression levels of miR-146b-5p and miR-21-5p were 3.02-fold and 2.75-fold higher, respectively, in tumors compared with normal tissues. In contrast, the miR-210-3p expression level in tumor tissue was only about one-third (0.33-fold) of the level detected in adjacent normal tissue.

**Figure 3:**
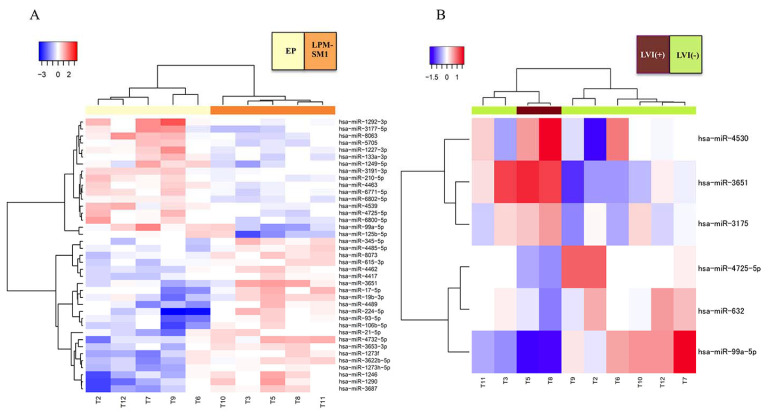
Unsupervised hierarchical clustering analysis of differentially expressed miRNAs in ESCC tissue according to invasion depth and lymphovascular invasion. (**A**) Patients were assigned to two groups based on depth of invasion (cluster A): epithelium (EP, *n* = 5) or lamina propria, muscularis mucosae, or submucosa (LP/MM/SM1, *n* = 5). See text for details. (**B**) Patients were assigned to two groups based on the presence (*n* = 2) or absence (*n* = 8) of lymphovascular invasion (Cluster B). Columns represent the patients and rows represent the individual miRNAs. Red and blue indicate high and low miRNA expression levels, respectively.

Next, we compared miRNA expression between the patient subgroups stratified by invasion depth and lymphovascular invasion. A total of 39 miRNAs were differentially expressed between tumor tissue derived from patients in the two invasion depth subgroups, of which 22 were upregulated and 17 were downregulated in the LP/MM/SM1 group (*n* = 5) compared with the EP group (*n* = 5) (Figure 3A, Supplementary Table S1). In contrast, only six miRNAs were differentially expressed between the two lymphovascular invasion subgroups, of which three were upregulated and three were downregulated in the group without lymphovascular invasion (*n* = 8) compared with the group with lymphovascular invasion (*n* = 2) (Figure 3B, Supplementary Table S2).

### 2.3. Validation of miR-21-5p and miR-146b-5p Expression in Superficial ESCC Tissues and Cell Lines

To validate the results of the miRNA array analysis, we performed qRT-PCR analysis of the 10 pairs of patient tissue samples and the human ESCC and normal esophageal cell lines. We selected miR-21-5p, miR-146b-5p, and miR-210-3p for this analysis because they were the three most differentially expressed miRNAs between ESCC tumor and adjacent normal tissues in the microarray analysis, and because insufficient total RNA was available for qRT-PCR analysis of additional miRNAs.

**Figure 4:**
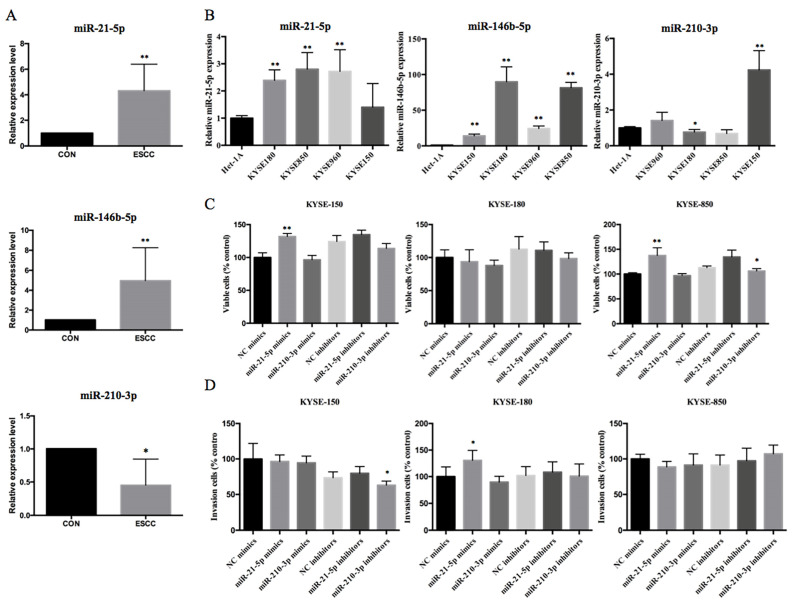
Validation of differential expression of miRNAs in ESCC tissues and cell lines. (**A**,**B**) qRT-PCR analysis of mi-21-5p, miR-210-3p, and miR-146b-5p in (**A**) 10 matched pairs of superficial ESCC compared with normal tissues (CON), and (**B**) ESCC cell lines compared with normal esophageal epithelial cells (Het-1A). ESCC cells were transfected with a control RNA or miR-21-5p, miR-210-3p, and miR-146b-5p mimics or inhibitors and analyzed in cell counting kit-8 (CCK-8) proliferations assays (**C**) or Transwell invasion and migration assays (**D**). Data are presented as the mean ± standard deviation of triplicates. * *p* < 0.05, ** *p* < 0.01 vs. control group.

Consistent with the microarray analysis, we found that miR-21-5p and miR-146b-5p were upregulated by 4.32-fold ± 2.08 (standard deviation) and 4.96-fold ± 3.30, respectively, and miR-210-3p was downregulated by 0.45-fold ± 0.06 in ESCC tissues compared with adjacent normal tissues (Figure 4A). Furthermore, the four ESCC cell lines KYSE150, KYSE180, KYSE850, and KYSE 960 also expressed significantly higher levels of miR-21-5p and miR-146b-5p and significantly lower levels of miR-210-3p compared with Het-1A normal esophageal epithelial cells (Figure 4B).

### 2.4. Overexpression or Inhibition of miR-21-5p and miR-146b-5p Expression Does Not Affect ESCC Cell Proliferation and Invasion

To explore the potential roles of miR-21-5p and miR-210-3p in ESCC, we transfected KYSE-150, KYSE-180, and KYSE-850 cells with miR-21-5p and miR-210-3p mimics or inhibitors and then assessed the effects on cell proliferation, migration, and invasion. As presented in Figure 4C,D, the proliferation and invasion of KYSE-180 cells transfected with miR-21-5p mimics were significantly increased compared with those of cells transfected with negative control (NC). These data suggest that overexpression of miR-21-5p promotes the cell proliferation and invasion of limited ESCC cells.

## 3. Materials and Methods

### 3.1. Patients and Samples

Paired samples of primary ESCC tissues and adjacent normal tissues were obtained from 12 patients who underwent esophageal ESD at Kagawa University Hospital between June 2017 and April 2018. Patients were referred for endoscopic resection after pathological diagnosis of stage I ESCC. None of the patients had received any treatment before sample collection. Two expert endoscopists (H.K. and N.N.) performed esophagogastroduodenoendoscopy with biopsy sampling according to a standardized protocol immediately before the patients underwent ESD. Paired tumor and adjacent normal tissue samples were taken from each patient. The closed biopsy forceps were passed through the working channel of the endoscope, positioned tangentially to the esophageal wall, and then opened. Using suction, the mucosa was pulled towards the tip of the endoscope and then grasped with biopsy forceps (Radial Jaw 4; Boston Scientific, Boston, MA, USA). All tissue samples were immediately frozen following resection and stored at −80 °C until analysis. ESCC tissues were confirmed histologically. The study was approved by the Institutional Review Board of Kagawa University (no. Heisei 22-063) and adhered to the ethical guidelines of the World Medical Association Declaration of Helsinki Ethical Principles for Medical Research Involving Human Subjects. Written informed consent for the use of their clinical samples and data was obtained from all patients.

### 3.2. Microarray Analysis of miRNA Expression

MiRNA array analysis was performed as described in our previous study [18]. Total RNA was extracted from tissue samples using a miRNAeasy Mini Kit (Qiagen, Hilden, Germany) according to the manufacturer’s instructions. MiRNA array was performed using a Human miRNA Oligo Chip (v. 21.0; Toray Industries, Tokyo, Japan). Total miRNA samples (200 ng) were labeled using a miRCURYHy3/Hy5 Power Labeling Kit, slides were scanned in a 3D-Gene Scanner 3000 (Toray Industries), and the fluorescence data were analyzed using 3D-Gene Extraction software (v. 1.2; Toray Industries). Quantile normalization was performed on raw data exceeding the background level, and differentially expressed miRNAs were identified by the Mann–Whitney U test. Hierarchical clustering was performed using the farthest end method, with the absolute non-central Pearson correlation coefficient as the metric. A heat map was created based on the relative expression intensity of each miRNA, using log2 of the fold change.

### 3.3. Quantitative Reverse Transcription-Polymerase Chain Reaction (qRT-PCR)

miR-21-5p, miR-210-3p, and miR-146b-5p were quantified in tissue samples and cell lines by qRT-PCR using human TaqMan MicroRNA Assay Kits (Applied Biosystems, Foster City, CA, USA), according to the manufacturer’s instructions. Primers and probes were also obtained from Applied Biosystems. RNU6B was evaluated as an internal control for the relative quantification of miRNAs, which was performed by the comparative cycle threshold (2^−ΔΔCT^) method, where ΔΔCT = ΔCt − average control ΔCt, and ΔCt = miRNA Ct − RNU6B Ct.

### 3.4. Cell Culture 

Five human ESCC cell lines (KYSE-150, KYSE-180, KYSE-850, and KYSE-960) were obtained from the Japanese Collection of Research Bioresources Cell Bank (Osaka, Japan). A non-tumorigenic human esophageal epithelial cell line (Het-1A) was obtained from the American Type Culture Collection (Manassas, VA, USA). KYSE-150, KYSE-180, KYSE-850, and KYSE-960 cells were cultured in Ham’s F12 (FujifilmWako Pure Chemical Corporation, Osaka, Japan) and RPMI-1640 (Gibco Invitrogen, Carlsbad, CA, USA) supplemented with 2% fetal bovine serum (533-69545; Wako, Osaka, Japan) and 100 mg/L penicillin-streptomycin (Invitrogen, Tokyo, Japan). All cells were maintained in a humidified 5% CO_2_ atmosphere at 37 °C.

### 3.5. Cell Transfection

miR-21-5p and miR-210-3p mimics and inhibitors and a control RNA sequence were obtained from Thermo Scientific (Waltham, MA, USA). KYSE150, KYSE180, and KYSE850 cells were seeded in six-well plates and grown for 24 h. The cells were then transfected with the appropriate miRNAs at a final concentration of 10 nM using Lipofectamine RNAiMAX (Invitrogen, Grand Island, NY, USA). After 24 h incubation, the cells were harvested, washed with ice-cold PBS, and used for experiments.

### 3.6. Cell Proliferation Assay

Cell proliferation was measured using the cell counting kit-8 (CCK-8) assay (Dojindo Laboratories, Kumamoto, Japan). Cells were seeded in 96-well plates at a density of 5 × 10^3^ cells/well and incubated for 24 or 48 h. CCK-8 reagent (10 µL) was then added to each well and the plates were incubated for an additional 2 h. Absorbance at 450 nm was measured with a microplate reader (Multiskan FC, Thermo Fisher Scientific, Tokyo, Japan).

### 3.7. Invasion Assays

Cell invasion was measured using a CytoSelect 96-well Cell Invasion Combo Assay Kit (basement membrane, colorimetric format; Cell Biolabs, San Diego, CA, USA) according to the manufacturer’s instructions. Invaded cells on the bottom of the membrane were stained and analyzed using a spectrophotometer.

### 3.8. Statistical Analyses

Data are expressed as the mean ± standard deviation (SD). All statistical analyses were performed using Prism 6 software (GraphPad Software, La Jolla, CA, USA). Nonparametric Wilcoxon/Mann–Whitney U-test was used to examine statistical significance between the two groups. A *p*-value less than 0.05 was considered significant.

## 4. Discussion

Early diagnosis of ESCC is challenging, due in large part to a lack of sensitive and specific biomarkers, and there is an urgent need for new early diagnostic tools. In this study, we performed a genome-wide miRNA analysis of superficial ESCC and paired adjacent normal tissues with the goal of identifying differentially expressed miRNAs that may serve as potential diagnostic markers for ESCC. We identified miR-21-5p and miR-146b-5p as two upregulated miRNAs and miR-210-3p as a significantly downregulated miRNA in ESCC tissues compared with matched normal tissues. To the best of our knowledge, this study is the first to report the miRNA expression profile of superficial ESCC.

In Japan, endoscopic diagnosis of invasion depth is usually classified into three categories: invasion limited to the EP or LP; invasion of the MM or submucosa to a depth of ≤200 µm (SM1); or invasion of the submucosa to a depth of >200 µM (SM2–3), because these categories correspond well with the risk of metastasis [19]. Whether or not endoscopic resection is indicated for superficial ESCC is based on the Esophageal Cancer Practice Guidelines 2017 [20]. However, there can be considerable discrepancy between clinical and pathological diagnoses, especially MM/SM1 cancers, because the accuracy of endoscopic diagnosis of pathological MM/SM1 is relatively poor [9,21]. Kato et al. reported diagnostic accuracies of 71% and 65% for superficial ESCC depth assessed by WLE alone and WLE with M-NBI endoscopy, respectively [7], while Wang et al. reported diagnostic accuracies of 53% and 57%, respectively, using the same two methods [8]. These studies emphasize the urgent need to identify biomarkers for superficial ESCC.

Dysregulation of miRNAs is associated with the development and progression of various cancers [11]. In the present study, we identified miR-21-5p and miR-146b-5p as significantly upregulated miRNAs in ESCC tumor tissues and cell lines. MiR-21-5p is known to be overexpressed in ESCC tissues [22,23] and can promote the growth, proliferation, invasion, and metastasis of ESCC cells by targeting multiple tumor suppressor genes, including programmed cell death protein 4 (PDCD4), PTEN, and Fas ligand (FasL) [24,25,26]. High expression of miR-21 is associated with shorter disease-free survival in several gastrointestinal malignancies [27,28]. Wang et al. analyzed the diagnostic value of several serum miRNAs and found that miR-21 had good sensitivity and specificity (71.0% and 96.9%, respectively) for discriminating between ESCC patients and healthy donors [28]. Although our results showing that miR-21-5p is overexpressed in early ESCC tissue are consistent with previous findings [22,23], we detect a partial effect of miR-21-5p overexpression on the malignant behaviors of KYSE-180 cells in vitro.

To clarify the functional role of differentially expressed miRNAs in ESCC, we analyzed the effects of miR-21-5p and miR-210-3p overexpression and inhibition on ESCC cell proliferation and invasion in vitro. Surprisingly, neither overexpression nor silencing of these miRNAs had any effects on these behaviors. Previous work showed that co-transfection of miR-203-3p and miR-21-5p mimics synergistically inhibited the proliferation, migration, and invasion of ESCC cells [29]. Thus, whether and how miR-21-5p, miR-146b-5p, and miR-210-3p affect ESCC cell malignancy remain to be clarified.

There are several limitations to our study. First, this was a retrospective study in a single institution. Second, the total number of patients is small, which further influenced the size of the patient subgroups. Third, the heterogeneity of the patients could have affected the miRNA analysis results. In addition, the differences in patient selection criteria, collection methods, and processing of biological samples may also contribute to the different miRNA signatures obtained. Finally, we did not elucidate the precise cellular origin of the differentially expressed miRNAs, and we cannot rule out the possibility that they were derived from non-tumor cells in the ESCC tissues. Further studies will be necessary to evaluate the identified miRNA signature in a larger cohort of patients with superficial ESCC, focusing on the variability in patients’ characteristics and experimental design.

In conclusion, this study identified significant differences in the miRNA expression profile of superficial ESCC tissues and adjacent normal tissues obtained before ESD, and several miRNAs were significantly associated with invasion depth and lymphovascular invasion. Further studies are warranted, not only to validate our results using a larger sample size, but also to investigate the mechanistic basis for differential miRNA expression. Our results suggest that differentially expressed miRNAs have potential utility as biomarkers of invasion depth and lymphovascular invasion in ESCC.

## Figures and Tables

**Table 1:** Patient characteristics.

Case	Sample No.	Sex	Age	Location	Tumor Size (mm)	Definitive Diagnosis	Clinical Diagnosis	Lymphovasular Invasion	Complication
1	T2	M	72	Mt	11 × 9	pT1a-EP	cT1a-EP	−	None
2	T3	F	77	Mt	45 × 24	pT1b-SM1	cT1a-SM1	−	None
3	T5	M	70	Ut	18 × 14	pT1a-LPM	cT1a-EP	+	None
4	T6	M	75	Mt	13 × 10	pT1a-EP	cT1a-EP	−	None
5	T7	M	73	Mt	10 × 9	pTa-EP	cT1a-EP	−	None
6	T8	M	62	Lt	38 × 20	pT1a-MM	cT1a-EP	+	None
7	T9	F	75	Mt	32 × 22	HGIN	cT1a-EP	−	None
8	T10	M	71	Mt	30 × 24	pT1a-MM	cT1a-LPM	−	None
9	T11	M	74	Mt	12 × 6	pT1a-LPM	cT1a-LPM	−	None
10	T12	M	82	Mt	17 × 8	pT1a-EP	cT1a-LPM	−	None

**Table 2:** Summary of significantly upregulated and downregulated miRNAs between superficial esophageal squamous cell carcinoma (ESCC) tumor and adjacent (non-tumor) tissues. Fold change (FC) > 2.0, FC < 0.5, *p*-value < 0.05.

miRNA	FC (Tumor/Non-Tumor)	*p*-Value	Chrosomal Location
Upregulated			
hsa-miR-223-3p	4.6	0.009	Xq12
hsa-miR-3687	3.15	0.002	
hsa-miR-146b-5p	3.02	0.003	10q24.32
hsa-miR-21-3p	2.97	0.003	17q23.1
hsa-miR-4716-3p	2.79	0.006	15q21.1
hsa-miR-21-5p	2.75	0.003	17q23.1
hsa-miR-4792	2.51	0.006	3p24.2
hsa-miR-711	2.39	0.007	3p21.31
hsa-miR-1273h-5p	2.2	0.001	16p12.1
hsa-miR-1273f	2.17	0.004	1p32.3
hsa-miR-6510-5p	2.13	0.002	17q21.2
hsa-miR-152-3p	2.1	0.01	17q21.32
hsa-miR-486-3p	2.05	0.001	
hsa-miR-7110-5p	2.02	0.009	3q21.1
Downregulated			
hsa-miR-210-3p	0.33	0.002	11p15.5
hsa-miR-203a-3p	0.35	0.003	14q32.33
hsa-miR-4688	0.39	0.007	11p11.2
hsa-miR-491-5p	0.4	0.005	9p21.3
hsa-miR-6851-5p	0.4	0.003	9p13.3
hsa-miR-513a-5p	0.42	0.009	Xq27.3
hsa-miR-6165	0.43	0.005	17q21.33
hsa-miR-6859-5p	0.44	0.002	1p36.33
hsa-miR-8078	0.44	0.001	18p11.32
hsa-miR-5006-5p	0.45	0.01	13q14.11
hsa-miR-4753-5p	0.48	0.006	1q42.3
hsa-miR-4655-3p	0.48	0.006	7p22.3
hsa-miR-4436a	0.49	0.006	2p11.2
hsa-miR-2392	0.49	0.005	14q32.2

## Data Availability

Data available upon request.

## Supplementary Materials

The following are available online at https://www.mdpi.com/article/10.3390/ijms22094789/s1, Table S1: Significantly differentially expressed miRNAs between EP and LPM/MM/SM1 superficial ESCC. Table S2: Significantly differentially expressed miRNAs between in lymphovasucular invasion (LVI) negative and positive.

## Abbreviations

ESCC	esophageal squamous cell carcinoma
miRNAs	microRNAs
WLE	white light endoscopy
M-NBI	magnifying narrow-band imaging
ESD	endoscopic submucosal dissection
EP	epithelium
LP	lamina propria
SM	submucosa
