# Supplementary material for: MicroRNA Expression Profiles in Superficial Esophageal Squamous Cell Carcinoma before Endoscopic Submucosal Dissection: A Pilot Study
Source: Int J Mol Sci. 2021 Apr 30;22(9):4789. doi: 10.3390/ijms22094789 (PMC8124636; doi:10.3390/ijms22094789)
Supplement: Supplementary file 1 [file ijms-22-04789-s001.zip › ijms-1179056-supplementary.pdf]

Supplementary Table S1. Significantly differentially expressed miRNAs between EP and LPM/MM/SM1 superficial ESCC. Fold change (FC)>2, FC<0.5, *P*-value<0.05.

miRNA	FC (LPM-SM1/ EP tumor)	*P*-value	Chrosomal location
**Upregulated**			
hsa-miR-1290	2.82	0.015	1p36.13
hsa-miR-224-5p	2.76	0.03	Xq28
hsa-miR-3687	2.57	0.009	
hsa-miR-3651	2.49	0.02	9q22.31
hsa-miR-4732-5p	2.47	0.011	17q11.2
hsa-miR-1246	2.47	0.019	2q31.1
hsa-miR-19b-3p	2.16	0.027	3q31.3
hsa-miR-4489	2.14	0.016	11q13.1
hsa-miR-17-5p	2.1	0.046	13q31.3
hsa-miR-106b-5p	2.05	0.024	7q22.1
**Downregulated**			
hsa-miR-1292-3p	0.39	0.033	20p13
hsa-miR-3177-5p	0.42	0.037	16p13.3
hsa-miR-99a-5p	0.44	0.036	21q21.1
hsa-miR-8063	0.47	0.016	15q14

Supplementary Table S2. Significantly differentially expressed miRNAs between in lymphovasucular invasion (LVI) negative and positive. Fold change (FC)>1.5, FC<0.67, *P*-value<0.05.

miRNA	FC (LVI positive/LVI negative)	*P*-value	Chrosomal location
**Upregulated**			
hsa-miR-3651	2.49	0.03	9q22.31
hsa-miR-4530	2.36	0.05	19q13.2
hsa-miR-3175	1.59	0.04	15q26.1
**Downregulated**			
hsa-miR-99a-5p	0.27	0.02	21q21.1
hsa-miR-4725-5p	0.51	0.04	17q11.2
hsa-miR-632	0.63	0.03	17q11.2
